# Cnidarian–algal partnerships structure bacterial communities during strobilation in *Cassiopea xamachana*

**DOI:** 10.1093/ismeco/ycag147

**Published:** 2026-06-05

**Authors:** Federica Montesanto, Mark McCauley, Samuel A Bedgood, Cody Miner, Bailey Steinworth, Victoria Sharp, Aki H Ohdera, Ayobami Oluokun, Mojibola Fowowe, Odunayo Oluokun, Yehia Mechref, Tingting Xiang, Mónica Medina, Virginia M Weis, Mark Q Martindale, Sandra Loesgen

**Affiliations:** Whitney Laboratory for Marine Bioscience, University of Florida, St. Augustine, FL 32080, United States; Department of Chemistry, University of Florida, Gainesville, FL 32611, United States; US Geological Survey, Wetland and Aquatic Research Center, Gainesville, FL 32653, United States; Department of Integrative Biology, Oregon State University, Corvallis, OR 97331, United States; Whitney Laboratory for Marine Bioscience, University of Florida, St. Augustine, FL 32080, United States; Department of Biology, University of Florida, Gainesville, FL 32611, United States; Whitney Laboratory for Marine Bioscience, University of Florida, St. Augustine, FL 32080, United States; Department of Biology, University of Florida, Gainesville, FL 32611, United States; Pennsylvania State University, State College, PA 16802, United States; Stony Brook University, Stony Brook, NY 11794, United States; Department of Chemistry and Biochemistry, Texas Tech University, Lubbock, TX 79409, United States; Department of Chemistry and Biochemistry, Texas Tech University, Lubbock, TX 79409, United States; Department of Chemistry and Biochemistry, Texas Tech University, Lubbock, TX 79409, United States; Department of Chemistry and Biochemistry, Texas Tech University, Lubbock, TX 79409, United States; Department of Bioengineering, University of California, Riverside, CA 92521, United States; Department of Ecology and Evolutionary Biology, University of California, Los Angeles, CA 90095, United States; Department of Integrative Biology, Oregon State University, Corvallis, OR 97331, United States; Whitney Laboratory for Marine Bioscience, University of Florida, St. Augustine, FL 32080, United States; Department of Biology, University of Florida, Gainesville, FL 32611, United States; Whitney Laboratory for Marine Bioscience, University of Florida, St. Augustine, FL 32080, United States; Department of Chemistry, University of Florida, Gainesville, FL 32611, United States

**Keywords:** Symbiodiniaceae, symbiosis, Scyphozoa, strobilation, holobiont, microbiome, 16S rRNA gene

## Abstract

Cnidarian–algal (Symbiodiniaceae) symbioses rely on complex interactions among the cnidarian host, algal symbionts, and associated bacterial communities. In the upside-down jellyfish *Cassiopea xamachana*, the polyp-to-medusa transition (strobilation) requires the establishment of symbiosis with Symbiodiniaceae algal partners, yet bacterial community dynamics during this developmental process remain unknown. Here, we experimentally induced symbiosis in aposymbiotic polyps using four algal treatments: xenic *Symbiodinium microadriaticum* (native symbiont), xenic *Breviolum minutum*, antibiotic-treated *B. minutum*, and a photosynthetically impaired *B. minutum* mutant. We combined 16S rRNA gene sequencing with measurements of photosynthetic efficiency, asexual budding, and algal surface N-glycan profiles to characterize holobiont assembly during symbiosis onset and strobilation. Algal treatment structured bacterial communities in both algal cultures and polyp tissues. Our analyses identified a set of amplicon sequence variants that consistently distinguished strobilating polyps from non-strobilating aposymbiotic and mutant polyps, in addition to potential bacterial biomarkers associated with successful metamorphosis. Strobilation was associated with the enrichment of bacterial communities putatively involved in sulfur and nitrogen cycling, whereas non-strobilating aposymbiotic and mutant polyps were characterized by opportunistic bacteria and increased community variability. Together, these results reveal coordinated changes in algal physiology, surface glycan profiles, and bacterial community structure associated with successful strobilation in *C. xamachana* and support a model in which tripartite host–alga–bacteria interactions influence cnidarian life stage transitions.

## Introduction

Metamorphic transitions in marine animals often require coordination among host developmental programs, environmental cues, and symbiotic partners [1–4]. In scyphozoan cnidarians, the transformation from a sessile polyp to a free-swimming medusa (strobilation) exemplifies this complexity: it involves segmental body division, nervous system remodeling, and, in some species, obligate symbiosis with intracellular dinoflagellates of the family Symbiodiniaceae [4–6]. Studies across diverse invertebrates have shown that associated microbes potentially contribute to settlement and metamorphosis via bioactive compounds [7–10]. Bacterial symbionts can influence life-history transitions by shaping local chemical environments, contributing metabolites, and regulating nutrient availability [9, 11–16]. In cnidarians, Symbiodiniaceae and associated microorganisms (bacteria, archaea, viruses, fungi) form a holobiont that acts as a single ecological and functional unit [13, 17–21]. Within this holobiont, bacterial symbionts contribute to nutrient cycling, defense, and stress resilience, and are increasingly recognized as key determinants of cnidarian phenotype and fitness [13, 17, 18, 20, 22–26]. Yet, how algal symbionts and their associated bacterial communities contribute to cnidarian developmental processes, particularly during key life-history transitions such as strobilation, remains poorly understood [22, 23, 27].

Microbiome studies on cnidarian life stages have revealed shifts in bacterial community composition during developmental transitions. In the moon jellyfish *Aurelia coerulea*, bacterial communities shift among polyps, ephyrae, and medusae, with stage-specific changes in dominant taxa [28–30]. In *Cassiopea*, larvae and medusae harbor distinct bacterial assemblages, with larvae enriched in *Pseudomonadota* and adult medusae often dominated by *Endozoicomonas* [31, 32]. Similar patterns of taxonomic variation among strains and locations, coupled with distinct bacterial assemblages, have been reported for the sea anemone *Exaiptasia* in both laboratory-maintained and wild populations [33, 34]. In *Cassiopea andromeda,* microbiomes contribute to holobiont nitrogen cycling, and symbiont loss is associated with *Vibrio*-dominated dysbiosis [35]. Beyond nutrient cycling, bacteria are increasingly recognized as active regulators of cnidarian developmental transitions. Recent work on *Cassiopea* settlement has identified specific bacterial taxa, particularly *Pseudoalteromonas* and *Vibrio* species, that can induce larval settlement and metamorphosis through biosynthetic pathways, though the exact molecular mechanisms remain unclear [10, 36]. Bacterial biofilms and algal-associated bacteria can produce settlement cues and signaling molecules [11, 37–40]. In cnidarian–algal symbioses, Symbiodiniaceae N-glycans play key roles in symbiont recognition and colonization, with stress-induced shifts toward high-mannose structures associated with reduced colonization [41, 42].

The upside-down jellyfish *Cassiopea xamachana* (hereafter referred to as *Cassiopea*) is an emerging model for dissecting these multi-partner interactions [6, 43–47]. *Cassiopea* can be maintained clonally and establishes stable symbioses with multiple Symbiodiniaceae (including *Symbiodinium microadriaticum*), relying on algal partners for carbon fixation and developmental cues; indeed, sessile polyps fail to strobilate without algal colonization [6, 43, 44, 46, 48]. These features highlight *Cassiopea* as a powerful model for dissecting host–alga–bacteria interactions, yet bacterial community composition and function during the polyp and strobilation stages remain poorly understood.

In this study, we investigate how algal symbiont identity and algal microbiome state shape bacterial community assembly in algal culture and *in hospite*, and how these communities are associated with strobilation timing and success. We used a simplified laboratory system consisting of clonally propagated aposymbiotic polyps, defined laboratory-maintained algal inocula, and a standardized *Artemia*-only feeding regime to test how algal treatment and associated bacterial communities covary with holobiont assembly and strobilation, in order to minimize host genetic and environmental variation and allow treatment-dependent effects to be resolved.

We inoculated the aposymbiotic polyps with four Symbiodiniaceae [49]: a xenic culture of the native symbiont *S. microadriaticum* (KB8), a xenic *Breviolum minutum* (SSB01) culture, an antibiotic-treated *B. minutum* culture, and a photosynthetically impaired *B. minutum* mutant *ORANGE 1* (*ora1*) [47], alongside an aposymbiotic control. Combining 16S rRNA gene amplicon sequencing with measurements of algal abundance, photosynthetic performance, algal N-glycan profiles, and asexual budding rate, we characterize how algal treatments structure holobiont-associated bacterial communities and identify features associated with successful metamorphosis.

## Material and methods

### Algal culturing and treatments

*Breviolum minutum* liquid culture (SSB01, confirmed by ITS2 sequencing; hereafter referred to as “Control”) was provided by Oregon State University and grown in Guillard’s (F/2) Marine Water Enrichment Solution (Millipore-Sigma G0154) prepared in artificial seawater (ASW; salinity before adding medium ~32 PSU). Algal cultures were incubated at 25–26°C on a 12 h light/12 h dark cycle with an irradiance of ~40 μmol photons m^−2^ s^−1^ of photosynthetically active radiation provided by NICREW white and blue LEDs.

An “antibiotic-treated” algal culture (hereafter referred to as “Antibiotic”) was generated by inoculating the *B. minutum* culture with penicillin–streptomycin (100 μg ml^−1^) and kanamycin (10 mg ml^−1^) for 1 month to reduce the bacterial component of the Symbiodiniaceae-associated microbiome [49].

Photosynthetically impaired algal cultures of *B. minutum* (*ora1*, confirmed by ITS2 sequencing; hereafter referred to as “Mutant”), generated by UV mutagenesis and characterized by loss of photosynthetic function including photosystem II activity [47], were grown on marine agar plates supplemented with 5 g l^−1^ glucose in continuous darkness at 25–26°C. The parental SSB01 line was originally established as a clonal, axenic algal culture. In this study, *ora1* was maintained under axenic culture conditions (sterile media and aseptic handling), but we did not independently re-establish or formally verify its axenicity; instead, we characterized its associated bacterial community using 16S rRNA gene amplicon sequencing.

Liquid culture of the native *S. microadriaticum* (KB8, confirmed by ITS2 sequencing; hereafter referred to as “Native”) was grown under the same conditions as *B. minutum* cultures at the Whitney Laboratory for Marine Bioscience.

### Experimental design

Aposymbiotic *C. xamachana* polyps from clonal line T1C were used in this study. They were obtained from a clonal colony that originated from a single aposymbiotic polyp collected from the Florida Keys, USA, that was allowed to bud continuously, forming a clonal colony. Aposymbiotic status was confirmed using epifluorescence microscopy (see below). Polyps were maintained in 0.2 μm-filtered ASW in small bowls at 25–27°C on a 12 h light/12 h dark cycle, fed whole freshly hatched *Artemia salina* three times per week, and subjected to water changes ~6 h after feeding for 45 days.

**Figure 1:**
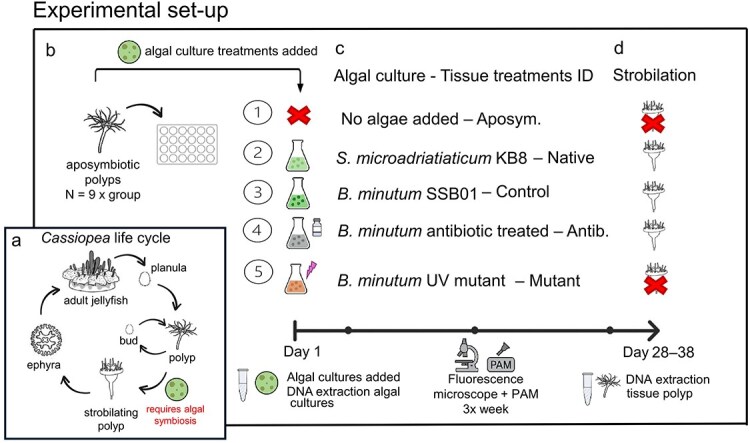
(a) Life cycle of *C. xamachana* illustrates the requirement for algal symbiosis to complete strobilation from polyp to ephyra stage. (b) Aposymbiotic polyps were maintained in 24-well plates prior to inoculation with algal treatments. (c) Five polyp treatments were examined (*N* = 9 per group), defined by the algal inoculation treatment: Aposymbiotic (polyps without algal symbionts), Native (polyps inoculated with *S. microadriaticum* KB8), Control (polyps inoculated with xenic *B. minutum* SSB01), Antibiotic (polyps inoculated with antibiotic-treated *B. minutum*), and Mutant (polyps inoculated with photosynthetically impaired *B. minutum ora1*). Treatment names refer to the polyps inoculated with these respective algal cultures. (d) Strobilation outcome. Timeline shows inoculation at Day 1, monitoring with fluorescence microscopy and PAM fluorometry throughout the experiment, and DNA extraction at inoculation for algal cultures and at strobilation for polyp treatments.

Prior to algal inoculation, all polyps were confirmed to be aposymbiotic by epifluorescence microscopy (Supplementary Fig. 1). Forty-five polyps were then transferred into transparent 24-well plates and simultaneously inoculated with ~1 × 10^6^ algal cells ml^−1^ per individual. Nine polyps were assigned to each of five polyp treatments (Native, Control, Antibiotic, Mutant, and Aposymbiotic; Fig. 1). Throughout this study, we use these treatment names (Native, Control, Antibiotic, Mutant, Aposymbiotic) as shorthand to refer to polyps inoculated with their respective algal culture treatments. That is, “Native” refers to polyps inoculated with *S. microadriaticum* KB8, “Control” refers to polyps inoculated with xenic *B. minutum* SSB01, “Antibiotic” refers to polyps inoculated with antibiotic-treated *B. minutum*, “Mutant” refers to polyps inoculated with the photosynthetically impaired *B. minutum ora1*, and “Aposymbiotic” refers to uninoculated control polyps maintained without algal symbionts. For the four symbiotic treatments, polyps were inoculated with their respective algal cultures (Native, Control, Antibiotic, or Mutant) using the same algal cell concentration and fed freshly hatched *A. salina* three times per week, with water changes ~6 h after feeding. An equal number of polyps were left uninoculated (Aposymbiotic treatment), transferred to a separate transparent 24-well plate, and fed identically. After 48 h, polyps in all treatments underwent a water change and were gently rinsed with ASW to remove any remaining free-living Symbiodiniaceae. Inoculation success was confirmed immediately by epifluorescence microscopy.

A single polyp was maintained in each well to allow precise tracking of individuals throughout the experiment. During the strobilation period (days 28–38), when the first and last strobilation events occurred, polyps from all treatments were sampled for DNA extraction. When the last polyp from the strobilating treatments (Native, Control, Antibiotic) strobilated (day 38), all remaining non-strobilated polyps (Aposymbiotic and Mutant) were sampled for DNA extraction (Fig. 1d). In parallel, an additional set of polyps inoculated with the Mutant algal culture was maintained for 60 days to test whether extended exposure would induce strobilation; these polyps were not included in the microbiome experiment and did not strobilate.

Throughout the experiment, asexual buds were identified on individual polyps, carefully removed, and placed in a separate container. Daily bud production was monitored for all treatments.

### Epifluorescence imaging for algal quantification

Symbiodiniaceae density in polyps (calyx and tentacles) was visualized with epifluorescence imaging, and each individual was tracked over the course of the experiment. We used a Zeiss Axioskop 2 FS fluorescence microscope to capture algal autofluorescence (Zeiss filter set: excitation 546/12 nm, dichroic 580 nm, emission long-pass 590 nm) with a Basler camera (model acA2240-35um). Imaging and quantification followed established protocols [42, 46, 47], with image analysis in ImageJ. Z-stack images were recorded for each individual and merged into a single projection, which was converted from color to greyscale to facilitate automated detection of algal cells. Algal cells in polyp were identified using focus stacking (z-stacks), and ImageJ was used to quantify algal abundance using the spot counter plugin, targeting the size (5–6 μm) and autofluorescence of algal cells (Supplementary Fig. 2).

### Photochemical efficiency of Symbiodiniaceae

Photosynthetic parameters were determined using a pulse-amplitude modulation (PAM) fluorometer (MINI-PAM-II, Walz GmbH, Germany). Effective quantum yield of PSII under actinic light (Y(II)) was measured twice daily (09:00 and 18:00), corresponding to the end of the dark and light phases, respectively. Before inoculation, all aposymbiotic polyps were measured in vivo at a constant distance (~10 mm) to confirm the absence of detectable photosynthetic signal. After inoculation, effective quantum yield of PSII (Y(II)), was measured daily throughout the experiment. The same parameters were recorded for each of the four photosynthetic algal cultures.

### Amplicon gene region sequencing

A total of 45 whole polyps (medusa plus stalk) were carefully picked from each well-plate, washed with 0.2 μm-filtered autoclaved artificial seawater to remove loosely associated microorganisms, and then individually homogenized using a pestle. DNA was extracted with the DNeasy PowerSoil kit (QIAGEN, CA, USA) following manufacturer’s instructions with few modifications (vortexing extended to 20 min, and final elution volume reduced to 25 μl). To characterize the bacterial communities associated with the algal inocula used in the experiment, aliquots from each algal culture treatment were collected at the time of inoculation and processed for 16S rRNA gene amplicon sequencing. To minimize cross-contamination between individuals, each polyp was maintained in a separate well throughout the experiment and all water changes were performed using 0.2 μm filtered autoclaved artificial seawater; homogenization tools were sterilized between samples. Six blank DNA extractions were processed in parallel with all samples to identify and account for any contaminant ASVs introduced during extraction, amplification, or sequencing. The 16S rRNA gene V4 hypervariable region was amplified using primer set 515F (Parada) (5′-GTGYCAGCMGCCGCGGTAA-3′) and 806R (Apprill) (5′-GGACTACNVGGGTWTCTAAT-3′) [50, 51]. The V4 hypervariable region of the 16S rRNA gene was selected because it is one of the most widely used and benchmarked regions for marine microbiome studies, and it has also been used in previous microbiome studies on cnidarians and *Symbiodiniaceae*-associated communities [32, 49]. PCR amplification was performed using the following conditions: initial denaturation at 95°C for 3 min, followed by 30 cycles of denaturation at 95°C for 30 s, annealing at 55°C for 30 s, and extension at 72°C for 30 s, with a final extension at 72°C for 5 min. Amplicon concentrations were confirmed with a Qubit 3 fluorometer (FisherSci), and quality was checked on a spectrophotometer (BioTek Take 3 Plate, Agilent) using the 260/280 nm ratio. DNA concentrations were normalized prior to sequencing. Libraries were sequenced at Genewiz (Azenta) on an Illumina MiSeq platform (2× 250 bp).

### Microbiome analysis

Sequences were filtered and trimmed using the DADA2 R-package (v1.22 [52]) in RStudio (2024.04.2 [53]), including quality filtering, error rate learning, denoising, merging, and chimera removal. Sequences were processed according to the DADA2 SOP (https://benjjneb.github.io/dada2/tutorial.html) using standard filtering (maxN = 0, maxEE = 2,2), denoising, merging, chimera and singletons removal, and naïve Bayesian taxonomic classification. A total of 3508 16S rRNA gene region amplicon sequence variants (ASVs) were identified across 75 samples. To assess and minimize contamination at multiple stages of the workflow, three types of negative controls were included: water blanks (0.2 μm filtered autoclaved artificial seawater), extraction, primers, and PCR blanks. Contaminant ASVs were identified and removed using the decontam R package (v3.19 [54]), applying the prevalence method with a threshold of 0.5, using extraction blanks as negative controls.

Taxonomic annotation of sequences was performed with the Silva database (v. 138.2 SSU [55]). ASVs not classified to the Kingdom “Bacteria”, or assigned as “Chloroplast” were removed. We rarefied to 6000 reads for alpha diversity and ordinations to facilitate comparability among treatments; differential abundance analyses were performed on unrarefied counts using DESeq2’s internal normalization. Sequence data and associated metadata were imported into phyloseq R-package (v1.50.0 [56]) for downstream analysis.

### Statistical analysis

All statistical analyses were conducted in RStudio (2024.04.2 [53]). For microbiome analyses, ASV tables and metadata were imported into phyloseq (v1.50.0 [56]) for data handling and visualization. After quality filtering and chimera removal (see Microbiome analysis), samples were rarefied to 6000 reads to standardize sequencing depth for alpha diversity and distance-based ordinations, excluding six low-depth samples. Four alpha diversity metrics were calculated using phyloseq: observed ASVs, Fisher’s alpha, Shannon index (H′), and Inverse Simpson index [57–60]. Treatment effects on alpha diversity were assessed with one-way analysis of variance (ANOVA) followed by Tukey’s honestly significant difference (HSD) post hoc tests for pairwise comparisons, implemented with rstatix (v0.7.2 [60]). Assumptions of normality and homogeneity of variances were evaluated using Shapiro–Wilk and Levene’s tests, respectively. When assumptions were violated, Kruskal–Wallis tests were used.

Community composition was analyzed using both Bray–Curtis dissimilarity and Weighted UniFrac distances. Distances were calculated in phyloseq and vegan (v2.6.10 [61]) from rarefied ASV tables and a phylogenetic tree constructed from aligned 16S rRNA gene sequences. Bray–Curtis dissimilarity and weighted UniFrac distances were calculated directly from rarefied ASV-level abundance matrices, preserving the full resolution of sequence-level diversity. Taxonomic agglomeration at family and genus levels was conducted independently using phyloseq and applied exclusively for compositional visualization. Differences in overall community structure among treatments were tested using permutational multivariate analysis of variance (PERMANOVA, 999 permutations) on these distance matrices. Homogeneity of multivariate dispersion was assessed, followed by permutation tests to evaluate differences in dispersion among groups. Principal coordinate analysis was used to visualize multivariate patterns, and 95% confidence ellipses were plotted around group centroids.

For outcome-based analyses, polyp samples were grouped according to strobilation status (Strobilation, Aposymbiotic, Mutant), and tests for differences in community composition (PERMANOVA) and homogeneity of variance/dispersion (betadisper) were repeated on these outcome groups. To facilitate taxonomic interpretation, ASVs were agglomerated to family and genus levels using phyloseq. Intersection analysis of ASV sharing across treatments (algae and polyp) was performed with ComplexUpset (v1.3.3 [62]), and summary metrics such as total richness and treatment-specific (unique) ASV counts were computed per group. Heat trees were constructed using metacoder (v0.3.8 [63]) after transforming counts to relative abundances (counts divided by total reads per sample × 100). Only taxa with a minimum of 5% total relative abundance across samples were retained to focus on prevalent community members, and for each substrate (polyp or algae) the most abundant taxa were selected based on total abundance.

Differential abundance at the ASV level among algae and polyp treatments was assessed using DESeq2 (v1.46.0 [64]) on unrarefied count data. DESeq2’s internal size-factor estimation and variance-stabilizing framework were used for normalization and dispersion estimation. Pairwise contrasts among treatments were extracted from the fitted models, and ASVs were considered differentially abundant if they had an absolute log2 fold-change above a biologically relevant threshold (typically |log2FC| ≥ 1) and a Benjamini–Hochberg false discovery rate (FDR)–adjusted *P* < .05. For analyses specifically targeting associations with developmental outcome (Strobilation vs. Aposymbiotic, Strobilation vs. Mutant), we used non-parametric Wilcoxon rank-sum tests on relative abundances at the ASV or genus level due to strong deviations from normality and zero inflation. ASVs were included in these tests if they were detected in at least 10% of samples across the two groups being compared, ensuring focus on prevalent community members while accounting for natural variation in colonization patterns. Multiple testing was again controlled using the Benjamini–Hochberg procedure; taxa were considered significantly associated with outcome if they met two criteria: (i) FDR-adjusted *P* < .05 and (ii) |Cohen’s *d*| > 0.2 [65], where Cohen’s *d* was computed from group means and pooled standard deviations to quantify effect size and direction. Significant taxa were visualized in heatmaps showing mean relative abundances across treatments or outcome groups, ordered by effect size.

For physiological and life-history traits, algal cell density trajectories were analyzed using linear mixed-effects models to account for repeated measurements through time. Models were fitted with treatment, time (days since inoculation), and their interaction as fixed effects and polyp identity as a random intercept term (using lme4 package v1.1.37 [66]), after inspection of residual diagnostics. When interactions were significant, we used estimated marginal means and Tukey-adjusted pairwise contrasts to compare treatments at specific time points. Photosynthetic efficiency (effective quantum yield of PSII, Y(II)) and bud production (total number of buds per polyp over the experiment) were analyzed among treatments using one-way ANOVA when assumptions were met; otherwise, Kruskal–Wallis tests were applied. When ANOVA or Kruskal–Wallis tests indicated significant treatment effects, we performed post hoc pairwise comparisons using Tukey HSD (for ANOVA) or Dunn’s test (for Kruskal–Wallis), with Benjamini–Hochberg correction. Figures were generated using ggplot2 (v4.0.0 [67]) and ComplexHeatmap (2.22.0 [68]).

### Glycan analysis

Algal samples (~1 × 10^7^ cells per sample) from each algal treatment (Native, Control, Antibiotic, and Mutant) were pelleted at 3100× *g*, the supernatant decanted, and pellets frozen. Samples were thawed and washed sequentially twice in 1 ml 2× PBS, 1 ml Milli-Q water, and 50 mM ammonium bicarbonate, pelleting cells between washes (14 000× *g* for 1 min). Cells were then treated with glycerol-free PNGase F (New England Biolabs, Rowley, MA, USA) under non-denaturing conditions following the manufacturer’s protocol to release N-glycans from cell surfaces. Pellets were incubated in 100 μl Glycobuffer 2, 900 μl ultrapure water, and 3 μl PNGase F at 37°C for 72 h with occasional inversion. After incubation, a few drops of HCl were added to stop the reaction, and samples were frozen and subsequently freeze-dried for 24 h. N-glycan cleanup was performed using C18 solid-phase extraction (SPE) cartridges with 5% acetic acid as the eluting solution. Eluted N-glycans were dried and subsequently reduced. Briefly, 10 μl of borane-ammonia complex (10 μg/μl) was added to each sample and incubated at 60°C for 1 h in a water bath. Methanol was added, and the mixture was dried (repeated four times) to remove residual borates. The reduced samples were then permethylated using the solid-phase approach as previously described [69, 70]. Briefly, sodium hydroxide beads (stored in DMSO) were loaded into an empty spin column and centrifuged at 1800 rpm for 2 min. The column was washed with 200 μl DMSO and centrifuged again. Glycan samples were dissolved in 30 μl DMSO, 1.2 μl NaOH, and 20 μl iodomethane, and then loaded onto the prepared spin column. Following a 25-min incubation at room temperature, an additional 20 μl of iodomethane was added, followed by a further 15-min incubation. The columns were centrifuged at 1800 rpm for 2 min. Permethylated glycans were eluted with 30 μl acetonitrile (ACN) and dried. Finally, samples were resuspended in 20% ACN containing 0.1% formic acid (FA) and centrifuged at 14800 rpm for 10 min prior to LC–MS/MS analysis. LC–MS/MS analysis was performed using a Dionex 3000 Ultimate Nano-LC system (Thermofisher Scientific, Sunnyvale, CA, USA) interfaced with an Orbitrap Fusion Lumos Tribrid Mass Spectrometer (Thermofisher Scientific, San Jose, CA, USA). Mobile phase A, which was made of 2% ACN, 98% HPLC-grade water, and 0.1% FA (v/v), and Mobile phase B, which was 100% ACN and 0.1% FA (v/v), were used. Glycans were separated on a reversed-phase Acclaim PepMap C18 column (150 mm × 75 μm i.d.) at 55°C with a flow rate of 0.35 μl/min. The 60-min multistep gradient was as follows: 20% mobile phase B (0–10 min), 42% B (10–10.001 min), 42%–55% B (10.001–50 min), 55%–90% B (50–50.001 min), 90% B (50.001–55 min), 90%–20% B (55–55.001 min), and 20% B (55.001–60 min).

MS analysis was conducted in positive ion mode with a full MS resolution of 120 000. The scan range was set at 400–2000 m/z with an isolation width of 2 m/z. The eight most intense precursor ions from the full MS scan were selected for CID MS/MS fragments. The MS2 resolution was set at 60 000, with a normalized collision energy (NCE) of 35%, an activation Q of 0.25, and an activation time of 10 ms. Data were initially processed using MultiGlycan software as previously described [71]. Peaks and their retention time for charge states +1 through +4 were then manually checked using Xcalibur (version 4.2, Thermofisher Scientific). Quantitative analysis was performed in Skyline (version 25.1.0.237, MacCoss Lab, University of Washington) to extract glycan abundances. Abundance values were subsequently normalized using probabilistic quotient normalization (PQN) [72].

## Results

### Algal-associated microbial community composition

**Figure 2:**
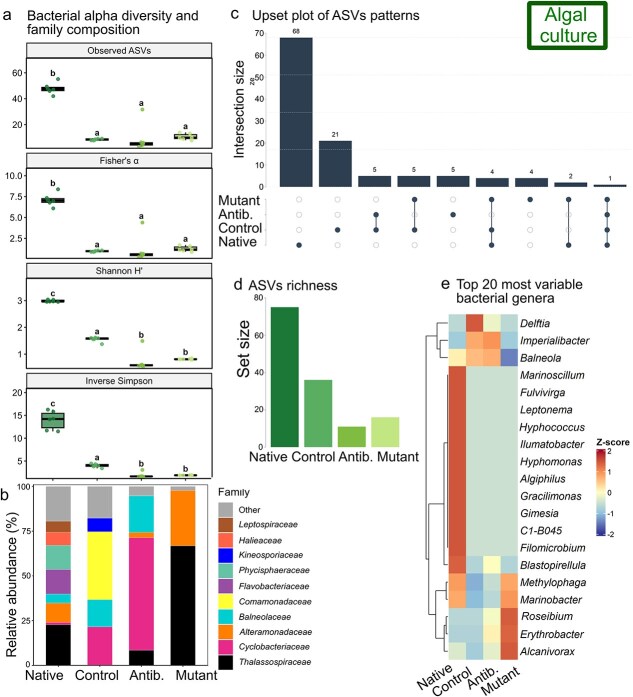
Bacterial (16S rRNA gene V4 region) community comparison between the four experimental treatment of algal cultures (Native, Control, Antibiotic, Mutant). (a) Alpha diversity metrics for richness, abundance, and evenness across experimental treatments. (b) Top 10 most abundant bacterial families across algal treatments. (c) UpSet plot illustrating ASV sharing patterns across algal treatments. Numbers above bars indicate intersection set sizes. (d) Total ASV richness per treatment. (e) Heatmap displaying the top 20 bacterial genera with highest variability across algal treatments, identified through differential abundance analysis.

Symbiodiniaceae-associated bacterial communities differed in alpha diversity among algal treatments (Fig. 2a). Native algal cultures supported the most diverse bacterial assemblages, with a mean of 47.83 ± 1.74 observed ASVs and a Shannon H′ of 2.98 ± 0.02, significantly higher than all *B. minutum* cultures for all metrics (Kruskal–Wallis, *P* < .001; Dunn’s test, *P* < .05 for all pairwise comparisons with Native). Within *B. minutum* treatments, Bmin cultures showed the lowest richness (mean 6.17 ± 0.17 ASVs, Shannon H′ 1.30 ± 0.02), followed by Control (8.50 ± 0.22 ASVs, Shannon H′ 1.55 ± 0.04). Antibiotic and Mutant cultures showed similarly low diversity (mean observed ASVs 9.33 ± 4.56 and 10.67 ± 0.95 respectively; Shannon H′ 0.72 ± 0.15 and 0.81 ± 0.01), significantly lower than both Native and Control cultures for Shannon and Inverse Simpson indices (Dunn’s test, *P* < .05). Antibiotic treatment did not reduce richness further relative to Bmin or Control cultures but altered community evenness, as reflected by the lower Shannon and Inverse Simpson values (mean InvSimpson 1.84 ± 0.25 vs 3.99 ± 0.15 in Control; Dunn’s test, *P* = .004).

Analysis of ASV sharing revealed treatment-specific bacterial communities with limited overlap (Fig. 2c and d). Native and Control hosted more unique ASVs than Antibiotic and Mutant, consistent with restructuring of bacterial assemblages. At the family and genus level, treatments showed distinct community compositions (Fig. 2b and e). Only one ASV was shared across all four algal culture treatments, yet this single ASV accounted for 24.4% of total reads, reflecting the highly treatment-specific nature of algal-associated bacterial communities. It should be noted that most dominant families in algal cultures are represented by a small number of ASVs (one to five ASVs per family), indicating that family-level patterns largely reflect the abundance of individual or few sequence variants rather than diverse within-family assemblages.

Native algal cultures were dominated by *Thalassospiraceae* (16.56 ± 2.43%), *Flavobacteriaceae* (13.76 ± 1.43%), *Phycisphaeraceae* (13.54 ± 1.23%), and *Alteromonadaceae* (10.82 ± 1.16%). Control algal cultures showed a distinct profile dominated by *Comamonadaceae* (44.42 ± 2.52%), *Cyclobacteriaceae* (24.93 ± 4.26%), and *Balneolaceae* (17.78 ± 1.01%). Control algal cultures displayed a further shift dominated by *Cyclobacteriaceae* (54.12 ± 2.06%) and *Balneolaceae* (22.20 ± 1.19%), with *Microbacteriaceae* (16.36 ± 2.60%) also prominent. Antibiotic algal cultures were dominated by *Cyclobacteriaceae* (62.88 ± 12.63%) and *Balneolaceae* (20.41 ± 4.25%), with high variability reflecting within-treatment heterogeneity. Mutant algal cultures were a unique composition dominated by *Thalassospiraceae* (66.78 ± 0.66%) and *Haliaceae* (30.91 ± 0.52%).

These differences in bacterial diversity are consistent with an effect of algal strain identity and the experimental manipulations applied, though contributions from other associated microbial sources cannot be fully excluded.

### Polyp-associated microbial community composition

All algal culture treatments, with the exception of the photosynthetic mutant, induced strobilation within 40 days (Fig. 1).

Community composition differed across *Cassiopea* polyps across all metrics (ANOVA, *P* < .001, for observed ASVs). Average within-sample richness was highest in Native polyps (mean 158.11 ± 9.29 ASVs), significantly higher than Aposymbiotic (98.33 ± 7.51; Tukey HSD, *P* = .001), Antibiotic (93.33 ± 12.39; *P* < .001), and Mutant polyps (77.14 ± 7.88; *P* < .001). Control polyps showed intermediate richness (133.11 ± 12.17), not significantly different from Native or Aposymbiotic polyps, but significantly higher than Mutant polyps (Tukey HSD, *P* = .006). Mutant polyps consistently showed the lowest richness and diversity across all metrics, with a mean Shannon H′ of 2.88 ± 0.08 and Inverse Simpson of 9.41 ± 0.72, significantly lower than both Native (Shannon H′ 3.82 ± 0.11, InvSimpson 22.24 ± 3.70; Dunn’s test, p = 0.004; Tukey HSD, *P* = .037) and Control polyps (Shannon H′ 3.71 ± 0.10, InvSimpson 23.36 ± 2.26; Dunn’s test, *P* = .007; Tukey HSD, *P* = .019). Antibiotic polyps exhibited high variability in Shannon diversity (3.13 ± 0.24), significantly lower than Native polyps (Dunn’s test, *P* = .033) but not significantly different from Aposymbiotic or Mutant polyps.

**Figure 3:**
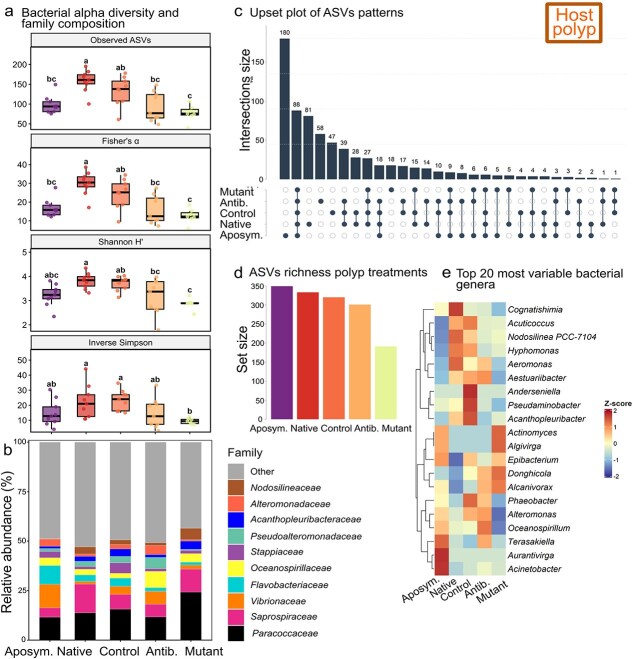
Bacterial (16S rRNA gene V4 region) community comparison among polyps inoculated with five different algal treatments (Native, Control, Antibiotic, Mutant) or maintained aposymbiotic. (a) Alpha diversity metrics for richness, abundance, and evenness across experimental treatments. (b) Top 10 most abundant bacterial families across polyp treatments. (c) UpSet plot illustrating ASV sharing patterns across polyp treatments. Numbers above bars indicate intersection set sizes. (d) Total ASV richness per treatment. (e) Heatmap displaying top genera of bacteria with highest variability across algal treatments, identified through differential abundance analysis.

Family-level taxonomic profiles differed among polyp treatments (Fig. 3b). *Paracoccaceae* was the dominant family across all treatments, reaching its highest relative abundance in Mutant polyps (26.69 ± 4.51%) and lowest in Antibiotic polyps (12.63 ± 2.38%). Aposymbiotic polyps were additionally characterized by elevated *Vibrionaceae* (12.49 ± 5.61%) and *Flavobacteriaceae* (10.32 ± 3.17%). Native polyps were distinguished by a high relative abundance of *Saprospiraceae* (16.38 ± 3.98%), which was lower in Control (8.16 ± 1.70%) and Antibiotic (6.92 ± 2.71%) polyps and nearly absent from Aposymbiotic polyps (5.09 ± 2.04%). Antibiotic polyps showed elevated *Oceanospirillaceae* (9.03 ± 3.55%) and *Vibrionaceae* (7.10 ± 3.83%), whereas Mutant polyps were characterized by the highest *Saprospiraceae* (12.26 ± 5.13%) and *Nodosilineaceae* (6.04 ± 4.14%).

UpSet analysis revealed that 180 ASVs were shared across all five polyp treatments, representing a common bacterial assemblage present regardless of algal symbiont identity, while each treatment also harbored treatment-specific ASVs (Fig. 3c and d). Of the 92 ASVs shared across all five polyp treatments, these accounted for 67.1% of total reads, indicating that while a relatively small number of ASVs constitute the shared core microbiome, they represent the numerically dominant fraction of the community. Concerning polyp-associated communities, dominant families were represented by a broader range of ASVs (*Paracoccaceae*: 46 ASVs; *Flavobacteriaceae*: 28 ASVs; *Cyclobacteriaceae*: 23 ASVs; *Saprospiraceae*: 12 ASVs), suggesting greater within-family diversity compared to algal culture communities. At the genus level, hierarchical clustering further resolved treatment-specific assemblages (Fig. 3e). Aposymbiotic polyps formed a distinct cluster enriched in *Terasakiella* and *Acinetobacter*. Native and Control polyps clustered together with partially divergent profiles. Antibiotic polyps were dominated by *Aestuariibacter* and *Oceanospirillum*, whereas Mutant polyps were enriched in *Algivirga* and *Alcanivorax*. While we cannot fully exclude microbial input from the food source, *A. salina* was hatched in 0.2 μm filtered autoclaved artificial seawater under standardized laboratory conditions. Importantly, the main treatment-specific taxa identified in this study were already detected in the algal cultures before *Artemia* was introduced, supporting the interpretation that the observed differences in community composition were primarily linked to the algal treatments. At the same time, we acknowledge that feeding may have influenced the abundance of some taxa, such as *Vibrio* and *Muricauda*, as *Artemia* can harbor chitin-associated bacteria, including *Vibrio* [49, 73, 74].

### Comparison of polyps and algal microbiomes

Bray–Curtis and weighted UniFrac ordinations revealed clear separation between polyp-associated and algae-associated bacterial communities across both distance metrics (PERMANOVA, *P* < .001 for all comparisons), indicating a strong effect of host substrate on community structure (Supplementary Fig. 3). Within polyp samples, Aposymbiotic and Mutant polyps consistently separated from symbiotic polyps, whereas Native, Control, and Antibiotic polyps formed distinct but partially overlapping groups. Differential abundance analysis identified treatment-specific enrichment of *Acinetobacter, Aurantivirga*, and *Brevundimonas* in Aposymbiotic polyps, *Aeromonas* and *Bythopirellula* in Native polyps, and *Bacillus* and *Algivirga* in Mutant polyps. Polyp-associated communities were broadly enriched in Bacteroidia and *Gammaproteobacteria*, whereas algae-associated communities showed enrichment of distinct *Bacteroidota* and *Planctomycetota* lineages.

### Treatment-specific taxa patterns in polyp microbiomes

**Figure 4:**
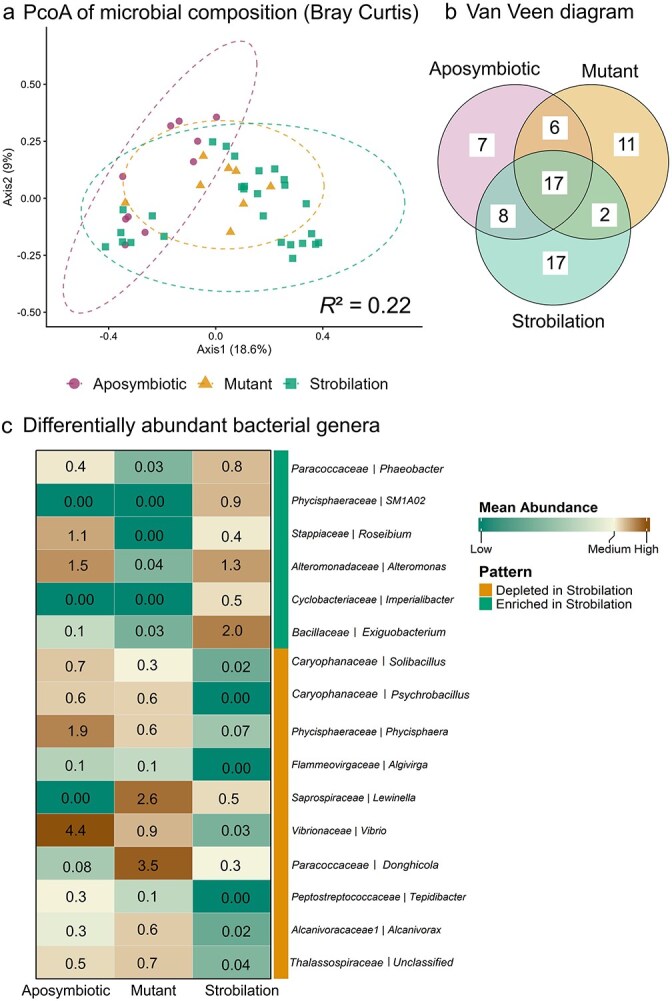
(a) Principal coordinates analysis of bacterial communities grouped by symbiotic outcomes: Strobilation (polyps inoculated with Native, Control, or Antibiotic algae that successfully strobilated), Mutant (polyps inoculated with photosynthetically impaired algae that failed to strobilate), and Aposymbiotic (uninoculated polyps). (b) Venn diagram of overlapping bacteria across the three groups. (c) Heatmap of bacterial genera that were most significantly different across Aposymbiotic, Mutant, and Strobilating groups, identified through differential abundance analysis.

To investigate bacterial community associations with successful polyp metamorphosis, polyp samples were grouped by developmental outcome into Strobilation (Native, Control, and Antibiotic treatments), Aposymbiotic (polyps not inoculated with algal symbionts) and Mutant (polyps inoculated with photosynthetically impaired algae that failed to strobilate). Bray–Curtis dissimilarity analysis revealed significant separation among these outcome groups (Fig. 4a; PERMANOVA, *R*^2^ = 0.22, *P* < .001), with Strobilation polyps differentiating from both Aposymbiotic and Mutant polyps along Axis 1 (18.6% of variance explained), whereas Aposymbiotic and Mutant samples showed only partial overlap, indicating related but non-identical non-strobilating community profiles.

Analysis of ASV sharing revealed that 17 ASVs were shared across all three outcome groups, representing a common assemblage present regardless of developmental outcome, while 17 ASVs were unique to the Strobilation group, 7 to the Aposymbiotic group, and 11 to the Mutant group (Fig. 4b). Mutant polyps shared only two ASVs exclusively with the Strobilation group, the lowest pairwise overlap among all outcome combinations, underscoring the compositional distinctiveness of communities associated with photosynthetically impaired symbiosis.

Pairwise differential abundance analyses identified a set of ASVs consistently distinguishing strobilating from non-strobilating polyps (Fig. 4c; Supplementary Fig. 4). Taxa enriched in strobilating polyps included *Cyclobacteriaceae* (*Imperialibacter*; mean relative abundance 0.5% in Strobilation vs 0.0% in both Aposymbiotic and Mutant polyps), *Bacillaceae* (*Exiguobacterium*; 2.0% vs 0.1% in Aposymbiotic and 0.03% in Mutant), *Flammeovirgaceae* (*Algivirga*; 0.0% in Strobilation vs 0.1% in Aposymbiotic), and *Caryophanaceae* (*Solibacillus*; 0.02% in Strobilation vs 0.7% in Aposymbiotic). In contrast, taxa depleted in strobilating polyps relative to non-strobilating groups included *Phycisphaeraceae* (*Phycisphaera*; 0.07% in Strobilation vs 1.9% in Aposymbiotic), *Stappiaceae* (*Roseibium*; 0.4% vs 1.1% in Aposymbiotic), *Paracoccaceae* (*Phaeobacter*; 0.8% vs 0.8% in Aposymbiotic and 0.03% in Mutant), and *Saprospiraceae* (*Lewinella*; 0.5% in Strobilation vs 2.6% in Mutant). Aposymbiotic polyps were additionally characterized by markedly elevated *Vibrionaceae* (*Vibrio*; mean 4.4% in Aposymbiotic vs 0.9% in Mutant and 0.03% in Strobilation), further distinguishing non-strobilating Aposymbiotic communities from those associated with successful metamorphosis.

Although sampling was conducted across a ten-day window tied to individual strobilation events, each polyp was sampled at its own metamorphic transition to ensure that microbiome profiles reflected the community state at the moment of strobilation rather than at an arbitrary time point; any residual temporal variance is therefore considered secondary to the biological signal of interest. While *Vibrio* can be introduced through *Artemia* feeding, we consider this unlikely to be an artifact of the feeding regime because *Vibrio* was present in algal cultures prior to feeding and is commonly found in polyps of wild *Cassiopea* [6, 31, 35, 36].

### Algal density *in hospite* and photosynthetic performance

**Figure 5:**
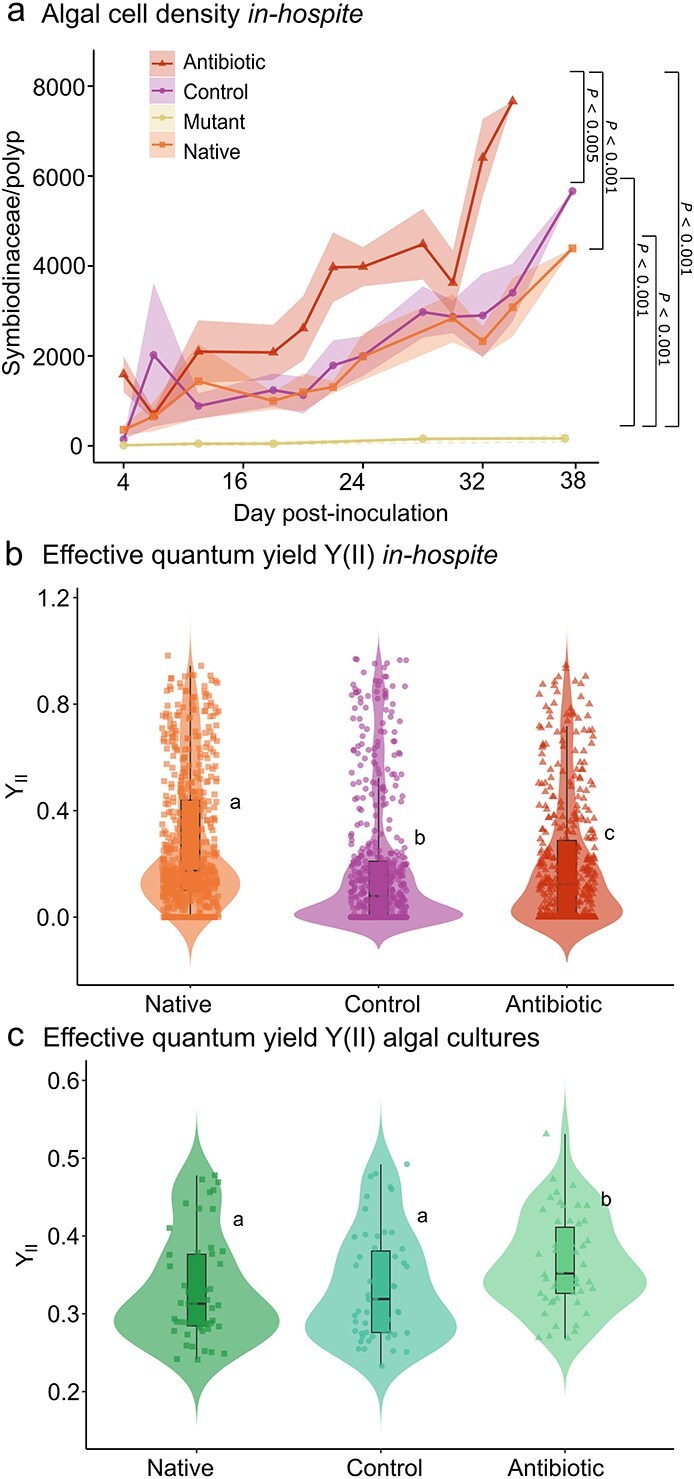
(a) Algal cell density dynamics in the polyps. (b) Effective quantum yield Y(II) measurements in hospite of polyp-associated symbionts. (c) Effective quantum yield Y(II) measurements of algal culture treatments.

Strobilating treatments showed sustained increases in algal cell density over the first 30 days, reaching peak densities of ~6000–8000 cells/polyp around the time of strobilation onset. Significant treatment effects on algal colonization dynamics were observed, with Control and Native treatments achieving successful colonization at similar rates, whereas the Antibiotic group reached strobilation faster and exhibited steeper increases in symbiont cell density. Mixed effects models with Tukey post hoc comparisons confirmed significant differences in colonization success between treatments (*P* < .05) (Fig. 5a).

Mutant polyps treatments maintained low algal densities throughout. Strobilation coincided with peak densities (Fig. 5a). No algal density was detected in aposymbiotic polyps.

Photosynthetic efficiency (Y(II)) varied little among algal cultures (Fig. 5c). *In hospite*, Native polyps exhibited significantly higher Y(II) than Control and Antibiotic (Kruskal–Wallis, *P* < .001; Fig. 5b). No signal was detected for Aposymbiotic or Mutant.

### Surface N-glycan profiles of algal cultures

**Figure 6:**
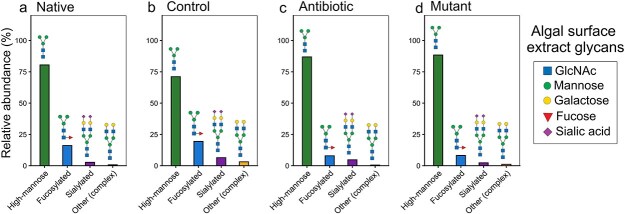
N-glycan composition of Symbiodiniaceae surface extracts across algal treatments. (a–d) Relative abundance of high-mannose, fucosylated, sialylated, and other (complex) N-glycans in cell-surface extracts from Native, Control, Antibiotic, and Mutant algal cultures, respectively.

Glycomic analysis revealed differences in N-glycan profiles (Fig. 6). All samples were dominated by high-mannose N-glycans, but Mutant and Antibiotic showed the strongest skew toward high-mannose structures, whereas Control displayed the lowest proportion and most complex glycans, with Native intermediate.

### Bud production

Aposymbiotic treatment showed the widest spread in bud production and included individuals with the highest numbers (>250 buds per polyp; Supplementary Fig. 5). However, median bud numbers in the Aposymbiotic treatment overlapped broadly with symbiotic treatments. Mutant polyps produced significantly fewer buds than Aposymbiotic polyps (Dunn’s post hoc, *P* < .05) but did not differ significantly from Native, Control, or Antibiotic treatments.

## Discussion

### Cross-kingdom interactions associated with holobiont assembly and strobilation

Bacterial community structure in both free-living algal cultures and polyps was strongly shaped by algal symbiont species and the bacterial communities associated with each algal culture, with treatment-specific effects explaining a substantial fraction of the observed variance. Differences among algal treatments are consistent with a strong influence of symbiont identity on bacterial recruitment in the phycosphere—the microbial environment immediately surrounding algal cells [37, 39] Within polyps, the influence of algal identity remained detectable but appeared to be modulated by host developmental state, with strobilating polyps showing distinct taxonomic profiles relative to non-strobilating ones. Polyp-associated bacterial communities were compositionally distinct from those of free-living algal cultures, suggesting that the polyp host imposes additional selective constraints on bacterial recruitment beyond those exerted by algal identity alone [13, 17, 18].

Differential abundance analyses comparing developmental outcomes identified a set of ASVs that consistently distinguished strobilating polyps from non-strobilating aposymbiotic and mutant polyps, representing candidate bacterial biomarkers associated with successful metamorphosis [3, 10]. Taxa enriched in strobilating polyps included *Cyclobacteriaceae* (*Imperialibacter*), which may contribute to polysaccharide degradation [71], and *Bacillaceae* (*Exiguobacterium*), a facultatively anaerobic lineage that may support stress tolerance. The presence of *Flammeovirgaceae* (*Algivirga*), an algae-associated taxon [37, 39], together with *Caryophanaceae* (*Solibacillus*), a spore-forming *Bacillota* [72], suggests that strobilation is associated with bacterial partners potentially capable of facilitating algal-polyp metabolic exchange and persisting through developmental transitions [3, 7, 9]. In contrast, several taxa were relatively depleted in strobilating polyps compared with non-strobilating ones, including *Phycisphaeraceae, Stappiaceae* (*Roseibium*), *Paracoccaceae* (*Phaeobacter*), and *Saprospiraceae* (*Lewinella*). *Vibrionaceae* (*Vibrio*) were nearly absent from successful strobilation treatments despite dominating aposymbiotic polyps, underscoring how symbiont presence versus absence restructures bacterial communities toward opportunistic, dysbiotic states [35, 75–80]. Although sampling was conducted across a 10-day window tied to individual strobilation events, each polyp was sampled at its own metamorphic transition to ensure that microbiome profiles reflected the community state at the moment of strobilation rather than at an arbitrary time point, and these patterns were observed under controlled laboratory conditions and may not fully reflect natural host-microbiome dynamics.

Our findings extend recent characterization of *Cassiopea* bacterial communities across life stages and algal culture conditions, which report taxonomic variation alongside broadly similar functional signatures [31]. Wild adult medusae host communities dominated by *Endozoicomonas* within diverse assemblages of *Pseudomonadota, Planctomycetota,* and *Bacillota* [31], whereas laboratory-maintained adult medusae host different communities dominated by *Moraxellaceae* and *Pseudomonadaceae* [75]. Here we add polyp-stage profiles: *Vibrio* dominance in aposymbiotic treatment, *Lewinella* enrichment in non-strobilating mutants, and functionally cohesive communities in strobilating polyps. Despite taxonomic differences across life stages and environments, a common pattern emerges: successful symbiotic states tend to coincide with relatively stable communities including lineages with putative roles in nitrogen cycling, whereas compromised symbioses are characterized by opportunistic taxa and greater community variability [75, 80, 81]. It should be noted that 16S rRNA gene amplicon sequencing resolves bacterial community composition and relative abundance but does not capture absolute bacterial load; future studies incorporating quantitative approaches such as 16S qPCR or spike-in normalization would provide a more complete picture of bacterial dynamics during strobilation. Taken together, these observations are consistent with the idea that cnidarian hosts may exert primarily functional, rather than strictly taxonomic, selection on their microbiomes, favoring bacterial partners that provide key metabolic capabilities important for developmental success and holobiont stability [12, 13, 17, 18, 32, 49, 75].

### Algal photosynthetic competence and bacterial community assembly

Species-specific differences in photosynthetic efficiency coupled with distinct bacterial signatures support the idea that algal strain identity may shape the phycosphere microenvironment through differential metabolite production [37, 39]. These findings align with studies showing that Symbiodiniaceae species harbor phylogenetically structured microbiomes [82] and that photosynthetic stress may alter algal surface glycan profiles [41], potentially disrupting the molecular recognition systems required for bacterial recruitment. The reduced bacterial diversity associated with photosynthetically impaired algae further supports a role for photosynthate availability in shaping holobiont community assembly.

Photosynthetic algae are known to release a substantial fraction of their fixed carbon as dissolved organic matter, creating a nutrient-rich phycosphere that can sustain diverse bacterial communities [39]. In contrast, the Mutant algal cultures cannot grow photoautotrophically and therefore lack photosynthetically derived carbon fixation [47], resulting in limited availability of endogenous organic carbon for both cellular metabolism and release to the surrounding phycosphere. This carbon limitation may place algal cells under metabolic stress and reduce the organic substrates available to associated bacteria. This constraint may help explain why mutant treatments did not recruit the nitrogen-cycling and other functionally important taxa associated with successful strobilation, as these organisms often require organic carbon inputs to fuel specialized metabolisms [71].

### Surface glycan composition and symbiosis

Mutant and Antibiotic algal treatments both exhibited elevated proportions of high-mannose N-glycans relative to Control ones. This pattern is consistent with work showing that heat stress increases high-mannose glycan abundance in *B. minutum* and is associated with reduced colonization efficiency [41], and with experiments in *Exaiptasia diaphana* where kifunensine-induced high-mannose enrichment impaired colonization success [42]. High-mannose structures are evolutionarily ancient and commonly linked to endoplasmic reticulum stress responses, and their enrichment in stressed symbionts has been proposed to reflect a more ancestral glycan state. Such glycomic shifts may reflect changes in symbiont quality that modulate host immune or recognition pathways in ways that reduce colonization success.

Complex N-glycans, including galactosylated and other terminal modifications, have been implicated as positive recognition signals for cnidarian hosts [83]. The reduced contribution of complex glycans (captured in the “Others” category) in Mutant and Antibiotic algal cultures, compared with Native and especially Control ones, is therefore consistent with the hypothesis that successful symbiosis depends on a balanced glycan profile rather than on simply high or low abundances of a single glycan class. Similar patterns of altered host reproduction with microbiome-manipulated symbionts occur in *E. diaphana* [49]. Overall, high-mannose enrichment does not appear to represent an absolute barrier to symbiosis, but may signal suboptimal symbiont quality that contributes to downstream effects on host physiology, resource allocation, and developmental competence.

### Metabolic trade-offs and holobiont fitness

Asexual budding patterns indicate that symbiotic state influences how *Cassiopea* polyps allocate resources to clonal propagation. Aposymbiotic polyps showed the widest spread in bud production, with some individuals producing high numbers of buds, although median bud numbers overlapped with those of symbiotic treatments. In contrast, Mutant polyps produced significantly fewer buds than all other groups, consistent with the idea that association with photosynthetically impaired algae represents a metabolically costly state that constrains investment in asexual reproduction without providing the developmental benefits of functional symbiosis. High variance in aposymbiotic budding may reflect differences in energetic status, whereas reduced Mutant budding suggests colonization by compromised algae imposes costs without providing developmental benefits [44, 84]. It should be noted, however, that these inferences are based on taxonomic composition data alone; few studies have directly characterized the active metabolism of cnidarian-associated bacteria using approaches such as metatranscriptomics or metaproteomics, and such methods will be essential to move beyond compositional inference toward functional validation. In *Exaiptasia*, antibiotic exposure and microbiome-altered Symbiodiniaceae impact host fitness and reproduction [49, 85]. In *Aurelia aurita*, antibiotic-mediated disruption reduced polyp survival and impaired strobilation over multiple generations [29]. Together with our results, these findings suggest that bacterial community structure contributes to fitness-related traits across diverse cnidarian lineages. In *Cassiopea*, successful strobilation was associated with host microbiomes that included lineages related to sulfur- and nitrogen-cycling taxa and *Bacillota*, whereas non-strobilating states were characterized by reduced diversity and, in the case of aposymbiotic polyps, *Vibrio* spp. dominance. Combined with previous work showing that successful symbiotic states tend to maintain nitrogen-cycling potential while stressed or bleached states often exhibit *Vibrio*-linked dysbiosis, these patterns are consistent with hosts favoring bacterial partners that provide key metabolic functions (especially in nitrogen cycling) rather than specific taxonomic identities [35, 75–80]. Glycomic data further show that Mutant and Antibiotic algal cultures are enriched in high-mannose glycans relative to Control, similar to stress-associated shifts described under thermal perturbation [41]. Such changes in algal surface chemistry may influence both bacterial recruitment and host recognition, linking symbiont physiology, microbiome assembly, and developmental outcomes. Our results identify specific host–alga–bacteria configurations that can be targeted in future studies to test their contributions to holobiont development and resilience.

## Conclusions

Together, the results presented here suggest that algal symbiont identity and associated bacterial communities contribute to shaping holobiont assembly in *C. xamachana*, with treatment-dependent effects detectable in both free-living algal cultures and polyp-associated microbiomes. The use of defined algal inocula in clonally propagated aposymbiotic polyps allowed treatment-dependent differences in bacterial community composition, alpha diversity, and developmental outcome to be resolved under controlled laboratory conditions, though whether these patterns generalize to natural host–symbiont assemblages remains to be tested.

Strobilation was associated with distinct bacterial community profiles relative to non-strobilating aposymbiotic and mutant polyps, with a set of consistently enriched ASVs representing candidate biomarkers of successful metamorphosis. These included lineages with putative roles in polysaccharide degradation and stress tolerance, whereas non-strobilating states were characterized by reduced diversity and, in the case of aposymbiotic polyps, dominance of opportunistic *Vibrionaceae*. Photosynthetic competence of the algal symbiont was further associated with bacterial diversity in hospite, glycan surface composition, and host investment in asexual reproduction, consistent with algal physiological state influencing holobiont assembly at multiple levels. The enrichment of high-mannose N-glycans in mutant and antibiotic-treated cultures relative to the xenic control is consistent with altered symbiont quality, though the functional consequences for bacterial recruitment remain to be established.

These observations are consistent with a model in which tripartite host–alga–bacteria interactions influence cnidarian life-stage transitions, with algal photosynthetic competence and phycosphere-associated microbiota among the factors contributing to holobiont community state and developmental outcome. Future work using functional approaches (e.g. including metatranscriptomics, proteomics, gnotobiotic reconstitution) could help to determine whether the bacterial taxa identified here actively contribute to strobilation or reflect the physiological state of the holobiont more broadly.

## Supplementary Material

Supplementary_material_ycag147

## Data Availability

Sequences are available under the BioProject submission PRJNA1390206. The complete analysis code is available on GitHub at https://github.com/federicamontesanto/Cassiopea_microbiome.git. The raw spectrum files are available on MassIVE database with ID: MSV000100426.
