# Supplementary material for: Cnidarian–algal partnerships structure bacterial communities during strobilation in Cassiopea xamachana
Source: ISME Commun. 2026 Jun 5;6(1):ycag147. doi: 10.1093/ismeco/ycag147 (PMC13298644; doi:10.1093/ismeco/ycag147)

Supplementary Figure 5. Asexual bud production across tissue treatment groups. (A) Temporal dynamics showing bud production over time. (B) Total number of buds produced per polyp. Violin plots show the distribution of values, with embedded boxplots indicating median and interquartile range and points representing individual polyps.

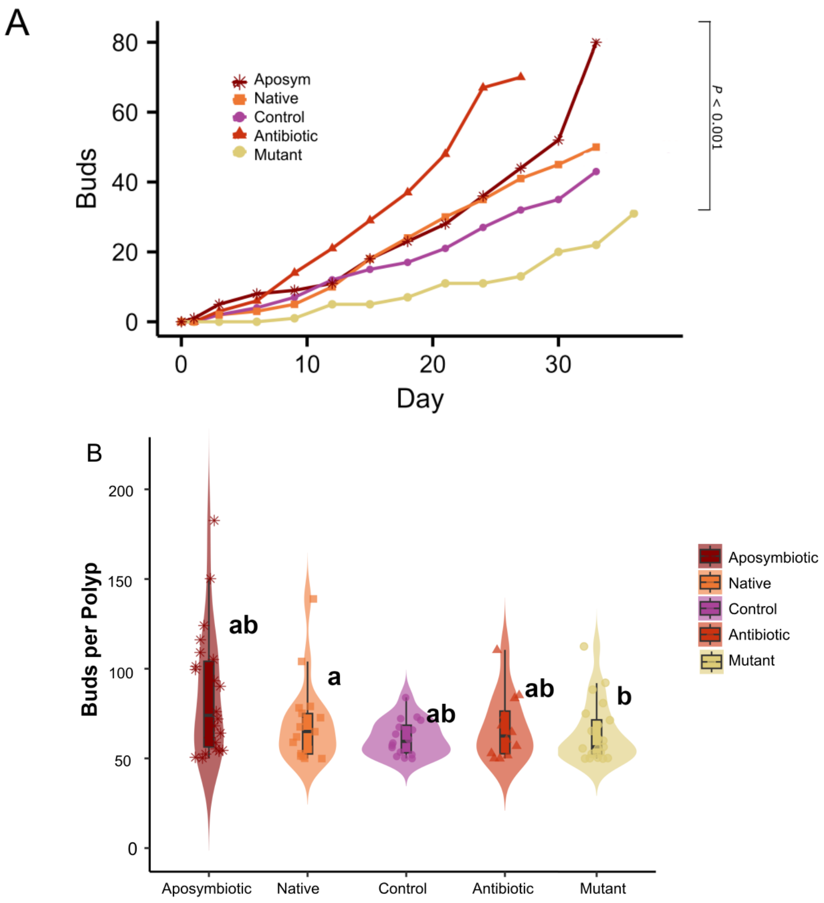

Supplement: Supplementary_material_ycag147 [file supplementary_material_ycag147.zip › Suppl_Fig5.pdf]
