# Supplementary material for: Cnidarian–algal partnerships structure bacterial communities during strobilation in Cassiopea xamachana
Source: ISME Commun. 2026 Jun 5;6(1):ycag147. doi: 10.1093/ismeco/ycag147 (PMC13298644; doi:10.1093/ismeco/ycag147)
Supplement: Supplementary_material_ycag147 [file supplementary_material_ycag147.zip › Supplementary Fig captions_2026_05_23.docx]

- Supplementary Figure 1. Pre-inoculation fluorescence screening of polyps to confirm aposymbiotic status. No fluorescence was detected in any polyp prior to inoculation, confirming their aposymbiotic status. Four representative polyps were photographed for each treatment: (A) aposymbiotic, (B) control, (C) antibiotic, (D) native, and (E) mutant.
- Supplementary Figure 2. Visualization and quantification of Symbiodiniaceae within *Cassiopea xamachana* polyps. (A) Polyp viewed under epifluorescence microscope with laser excitation inducing algal autofluorescence. (B) Z-stack merged image converted to grayscale for analysis. (C) ImageJ analysis showing enumerated cells for density calculations.
- Supplementary Figure 3. Principal Coordinates Analysis of algal cultures and polyp-associated bacterial communities. (A) Bray-Curtis dissimilarity and (B) Weighted UniFrac distances of polyps inoculated with different algal treatments. Ellipses represent 95% confidence intervals. (C) Bray-Curtis dissimilarity and (D) Weighted UniFrac distances between polyp and algal treatments. (E) Genus-level heatmap showing differential abundance between polyphost and algal samples. (F) Heat trees displaying the phylogenetic relatedness and taxonomic diversity of bacteria enriched in polyp treatments. (G) Heat trees displaying the phylogenetic relatedness and taxonomic diversity of bacteria enriched in algal treatments.
- Supplementary Figure 4. Bacterial taxa showing significant differential abundance between strobilation and aposymbiotic polyps or between strobilation and mutant polyps, displayed as Cohen's d effect sizes.
- Supplementary Figure 5. Asexual bud production across polyp treatment groups. (A) Temporal dynamics showing bud production over time. (B) Total number of buds produced per polyp. Violin plots show the distribution of values, with embedded boxplots indicating median and interquartile range and points representing individual polyps.
