# Supplementary material for: Cnidarian–algal partnerships structure bacterial communities during strobilation in Cassiopea xamachana
Source: ISME Commun. 2026 Jun 5;6(1):ycag147. doi: 10.1093/ismeco/ycag147 (PMC13298644; doi:10.1093/ismeco/ycag147)

Supplementary Figure 1. Pre-inoculation fluorescence screening of polyps to confirm aposymbiotic status. No fluorescence was detected in any polyp prior to inoculation, confirming their aposymbiotic status. Four representative polyps were photographed for each treatment:
(A) aposymbiotic, (B) control, (C) antibiotic, (D) native, and (E) mutant.

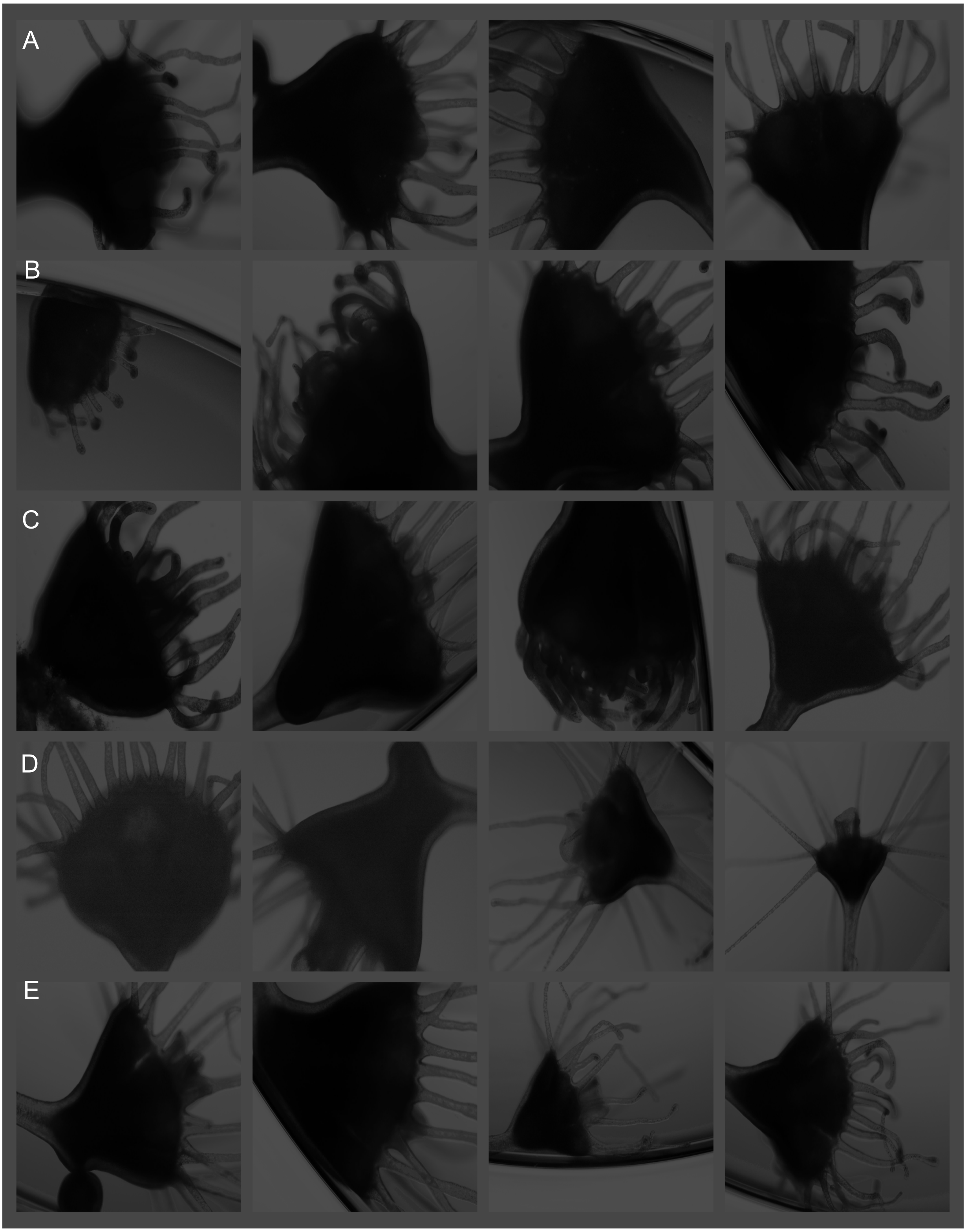

Supplement: Supplementary_material_ycag147 [file supplementary_material_ycag147.zip › Suppl_Fig1.pdf]
