# Supplementary material for: Cnidarian–algal partnerships structure bacterial communities during strobilation in Cassiopea xamachana
Source: ISME Commun. 2026 Jun 5;6(1):ycag147. doi: 10.1093/ismeco/ycag147 (PMC13298644; doi:10.1093/ismeco/ycag147)

Supplementary Figure 2. Visualization and quantification of Symbiodiniaceae within *Cassiopea xamachana* polyps. (A) Polyp viewed under epifluorescence microscopy with laser excitation inducing algal auto-fluorescence. (B) Z-stack merged image converted to grayscale for analysis. (C) ImageJ analyis showing enumerated cells for density calculations.

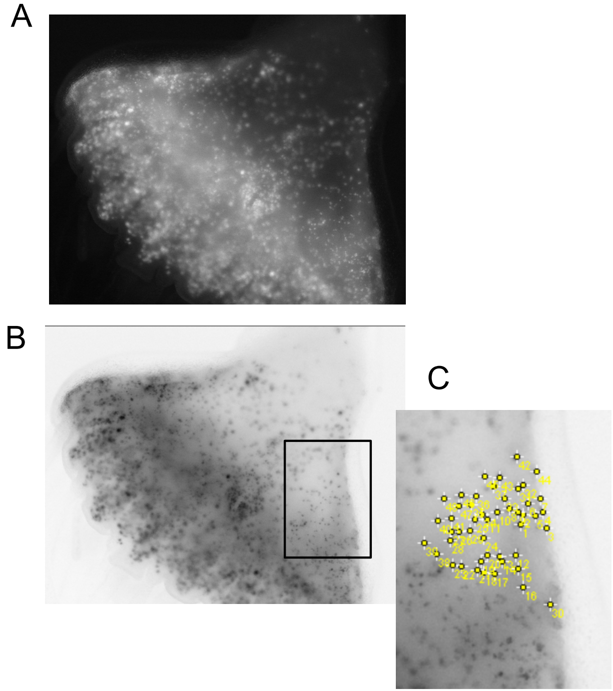

Supplement: Supplementary_material_ycag147 [file supplementary_material_ycag147.zip › Suppl_Fig2.pdf]
