# Supplementary material for: Cnidarian–algal partnerships structure bacterial communities during strobilation in Cassiopea xamachana
Source: ISME Commun. 2026 Jun 5;6(1):ycag147. doi: 10.1093/ismeco/ycag147 (PMC13298644; doi:10.1093/ismeco/ycag147)

Supplementary Figure 3. Principal Coordinates Analysis of algal cultures and polyp-associated bacterial communities. (A) Bray-Curtis dissimilarity and (B) Weighted UniFrac distances of polyps inoculated with different algal treatments. Ellipses represent 95% confidence intervals. (C) Bray-Curtis dissimilarity and (D) Weighted UniFrac distances between polyp and algal treatments. (E) Genus-level heatmap showing differential abundance between polyphost and algal samples. (F) Heat trees displaying the phylogenetic relatedness and taxonomic diversity of bacteria enriched in polyp treatments. (G) Heat trees displaying the phylogenetic relatedness and taxonomic diversity of bacteria enriched in algal treatments.

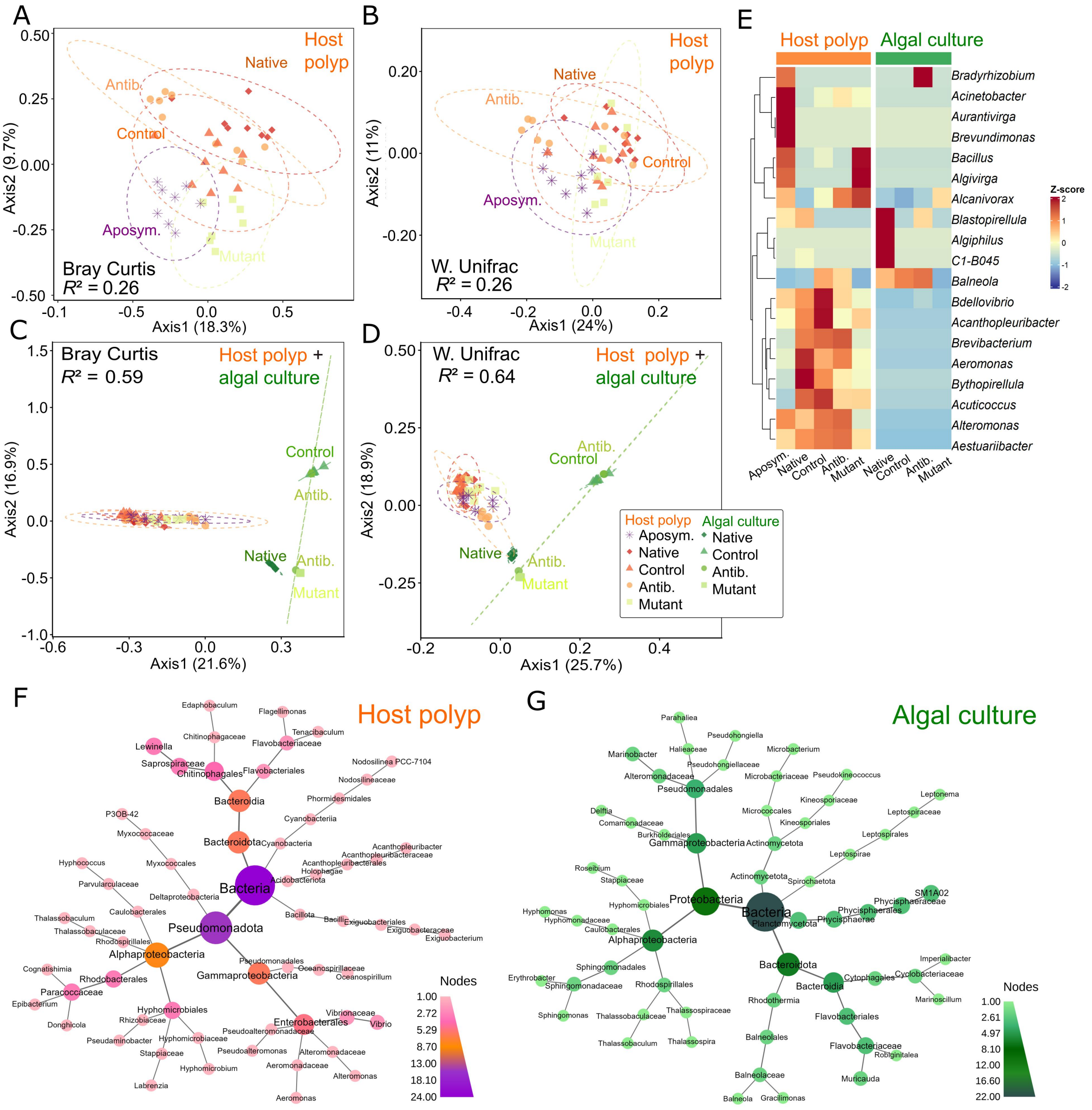

Supplement: Supplementary_material_ycag147 [file supplementary_material_ycag147.zip › Suppl_Fig3.pdf]
