# Supplementary material for: Cnidarian–algal partnerships structure bacterial communities during strobilation in Cassiopea xamachana
Source: ISME Commun. 2026 Jun 5;6(1):ycag147. doi: 10.1093/ismeco/ycag147 (PMC13298644; doi:10.1093/ismeco/ycag147)

Supplementary Figure 4. Bacterial taxa showing significant differential abundance between strobilation and aposymbiotic polyps or between strobilation and mutant polyps, displayed as Cohen's d effect sizes.

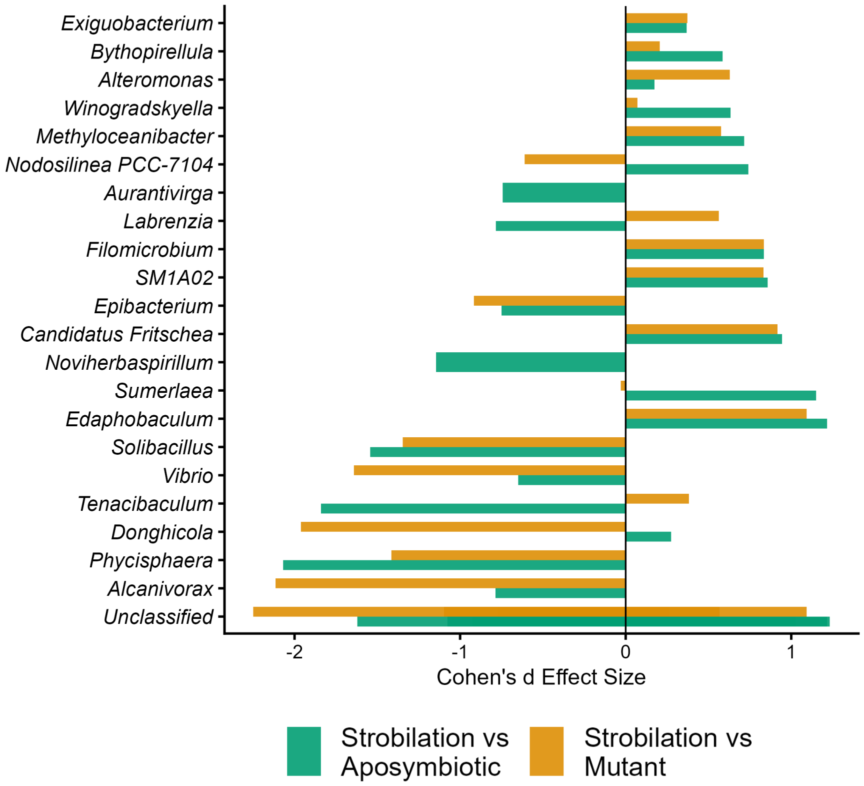

Supplement: Supplementary_material_ycag147 [file supplementary_material_ycag147.zip › Suppl_Fig4.pdf]
